# Exome sequencing identifies titin mutations causing hereditary myopathy with early respiratory failure (HMERF) in families of diverse ethnic origins

**DOI:** 10.1186/1471-2377-13-29

**Published:** 2013-03-20

**Authors:** Camilo Toro, Montse Olivé, Marinos C Dalakas, Kumaraswami Sivakumar, Juan M Bilbao, Felix Tyndel, Noemí Vidal, Eva Farrero, Nyamkhishig Sambuughin, Lev G Goldfarb

**Affiliations:** 1 Undiagnosed Diseases Program, National Human Genome Research Institute, National Institutes of Health, Bethesda, MD, USA; 2 Institute of Neuropathology, Department of Pathology and Neuromuscular Unit, Department of Neurology, IDIBELL-Hospital Universitari de Bellvitge and Centro de Investigación Biomédica en Red de Enfermedades Neurodegenerativas (CIBERNED), Barcelona, Spain; 3 University of Athens Medical School, 75 Mikras Asias, Athens, Greece; 4 Barrow Neurological Institute, Phoenix, AZ, USA; 5 Sunnybrook Health Sciences Centre, University of Toronto, Toronto, ON, Canada; 6 Toronto Western Hospital, University of Toronto, Toronto, ON, Canada; 7 Institute of Neuropathology, Department of Pathology, IDIBELL-Hospital Universitari de Bellvitge, Barcelona, Spain; 8 Pneumology Service, IDIBELL-Hospital Universitari de Bellvitge, Barcelona, Spain; 9 Uniformed Services University, Bethesda, MD, USA; 10 National Institute of Neurological Disorders and Stroke, National Institutes of Health, Room 4S06, 5625 Fishers Lane, Bethesda, MD MSC 9404, USA

**Keywords:** Myopathy, Respiratory failure, HMERF, Titin, Titin A-band mutation, Fibronectin type III

## Abstract

**Background:**

Hereditary myopathy with early respiratory failure (HMERF) was described in several North European families and recently linked to a titin gene (*TTN*) mutation. We independently studied HMERF-like diseases with the purpose to identify the cause, refine diagnostic criteria, and estimate the frequency of this disease among myopathy patients of various ethnic origins.

**Methods:**

Whole exome sequencing analysis was carried out in a large U.S. family that included seven members suffering from skeletal muscle weakness and respiratory failure. Subsequent mutation screening was performed in further 45 unrelated probands with similar phenotypes. Studies included muscle strength evaluation, nerve conduction studies and concentric needle EMG, respiratory function test, cardiologic examination, and muscle biopsy.

**Results:**

A novel *TTN* p.Gly30150Asp mutation was identified in the highly conserved A-band of titin that co-segregated with the disease in the U.S. family. Screening of 45 probands initially diagnosed as myofibrillar myopathy (MFM) but excluded based on molecular screening for the known MFM genes led to the identification of a previously reported *TTN* p.Cys30071Arg mutation in one patient. This same mutation was also identified in a patient with suspected HMERF. The p.Gly30150Asp and p.Cys30071Arg mutations are localized to a side chain of fibronectin type III element A150 of the 10th C-zone super-repeat of titin.

**Conclusions:**

Missense mutations in *TTN* are the cause of HMERF in families of diverse origins. A comparison of phenotypic features of HMERF caused by the three known *TTN* mutations in various populations allowed to emphasize distinct clinical/pathological features that can serve as the basis for diagnosis. The newly identified p.Gly30150Asp and the p.Cys30071Arg mutation are localized to a side chain of fibronectin type III element A150 of the 10th C-zone super-repeat of titin.

## Background

Hereditary myopathy with early respiratory failure (HMERF) also known as Edström myopathy is a disorder manifesting with predominantly proximal muscle weakness of the lower and upper extremities with respiratory insufficiency and involvement of neck flexors early in the disease course [1]. A number of cases showing autosomal dominant pattern of inheritance and sporadic cases have been reported [2-4]. A distinctive pathological feature of HMERF is the presence of cytoplasmic bodies. The first *TTN* mutation causing HMERF has been identified by Lange and colleagues [5] in Swedish families originally described by Edström et al. [1]. This mutation was defined as p.Arg279Trp by using residue numbering according to the crystal structure of the titin kinase domain [6]. Based on updated databases (GenBank NP_001243779 and UniProt Q8WZ42), the mutant residue has been re-numbered as p.Arg32450Trp [7]. Recently, HMERF in several newer North European families have been associated with a g.274375T>C: p.Cys30071Arg mutation in the A-band of titin [7,8].

*TTN* mutations have been known to cause other neuromuscular and cardiac disorders, among them dilated cardiomyopathy type 1G [9] and neuromuscular disorders such as tibial muscular dystrophy (TMD), or Udd myopathy [10], limb–girdle muscular dystrophy type 2J (LGMD2J) [11,12], and autosomal recessive early-onset myopathy with fatal cardiomyopathy (EOMFC) [13]. Mutations associated with dilated cardiomyopathy are overrepresented in the titin A-band [14]; the mutation identified in Swedish HMERF families by Lange et al. [5] is in the titin protein kinase domain, while mutations for TMD, LGMD2J, and EOMFC are located in the C-terminal end of the M-band.

Titin is the largest muscle protein known, a filamentous molecule stretching for half-sarcomere, from the Z-disk (N-terminus) spanning the A-band and extending to the M-band (C-terminus) [15]. Titin isoform of skeletal muscle is composed of >33,000 amino acids, weighs 3,700 kD, and its length is 2 μm [16]. Titin has a modular structure; up to 90% of its mass consists of repeating immunoglobulin-like (Ig) and fibronectin type III (FN3) domains. Titin serves as a molecular template for the assembly of the myosin-based filament and is responsible for the stabilization of the thick filament and the structural integrity of the entire sarcomere by acting as a scaffold [17]. Titin is a molecular spring that provides elasticity to the sarcomere and ensures its return to the original length after muscle relaxation [18]. Titin is also involved in signal transduction from the myofibrils to other compartments of the muscle cell, including the nucleus [19].

The elastic part of titin is its I-band region composed of 40 Ig-domains; the A-band is stable and not extensible due to its strong interaction with the thick filament [20,21]. The A-band is composed of stretches of FN3 domains interspaced by single Ig domains, forming the unique titin super-repeat architecture. The N-terminal super-repeat within the A-band (D-zone) comprises six copies of a 7-element structure arranged as Ig-(FN3)2-Ig-(FN3)3. The second super-repeat located C-terminally (C-zone) is organized into eleven copies of an 11-element motif arranged as Ig-(FN3)2-Ig-(FN3)3-Ig-(FN3)3 [22]. The two super-repeats of the A-band region provide regularly spaced binding sites for components of the thick filaments [17,23,24] and serve as a molecular ruler that regulates the assembly and the length of the thick filament [25]. FN3 elements provide binding sites for myosin, while Ig-like domains may be responsible for interaction with other ligands. A-band is evolutionarily conserved, unlike the Z-disk and I-band segments of titin that are highly divergent [18]. Titin kinase domain contains a catalytic domain and an auto-regulatory C-terminal tail [6], which wraps the active site of the catalytic domain. The p.Arg32450Trp mutation is located at the N-terminal helix (alphaR1) of the kinase domain [5]. *TTN* gene is positioned in the 2q31 chromosome region and consists of 363 exons.

In the process of genetic testing of patients with myofibrillar myopathy (MFM) we encountered familial and sporadic cases of skeletal myopathy with or without associated respiratory abnormalities who lack mutations in MFM-associated genes [26,27]. To identify a causative mutation in affected members of a large U.S. family suffering from proximal myopathy and respiratory failure we carried out whole exome sequencing and determined that the mutation was in the *TTN* gene. Screening of other families led to the identification of a similar mutation in affected individuals from two other families originating from a Native American population in Canada and from Spain, indicating that missense mutations in *TTN* are the cause of HMERF in families of divergent origins. We compared phenotypic features of HMERF in three families under our study with previously reported clinical/pathological descriptions of the disease caused by three *TTN* mutations in various populations and refined diagnostic criteria of HMERF. Both p.Gly30150Asp and p.Cys30071Arg *TTN* mutations disrupt a fibronectin type III element of titin A-band super-repeat designated as A150.

## Methods

### Clinical and laboratory evaluation

We studied in detail 7 patients from three unrelated families reffered to as families A, B, and C suffering from skeletal myopathy and respiratory failure. Studies included muscle strength evaluation according to the Medical Research Council (MRC) grading scale, serum CK level assessment, nerve conduction studies and concentric needle EMG, respiratory function test, and cardiologic examination. In one patient muscle CT scan images were obtained from the pelvis, mid-thigh and mid-calf levels. Written consent was obtained for each element of this study. Genetic studies were approved by the Institutional Review Board of the National Institute of Neurological Disorders and Stroke in Bethesda, Maryland.

### Muscle biopsy

An open muscle biopsy was performed for diagnostic purposes in at least one affected patient per family. Muscle tissue was obtained from the deltoid, biceps, quadriceps or gastrocnemius muscles. Biopsies were taken one to 14 years after the initial symptoms. The samples were immediately frozen in liquid nitrogen-cooled isopentane and processed for routine histochemical reactions. Immunocytochemical analysis was performed using antibodies against desmin and vimentin in 4 patients of family A and myotilin, dystrophin, αB-crystallin, and filamin C in a patient of family C as previously described [27]. In addition, muscle sections from the proband of family B were processed for TDP43 immunohistochemistry using a rabbit polyclonal (ProteinTech, Chicago, Ill). Pieces of biopsied tissue from the proband of family B and C were processed for ultrastructural examination using standard methods.

### Whole exome sequencing and SNP analysis

**Figure 1:**
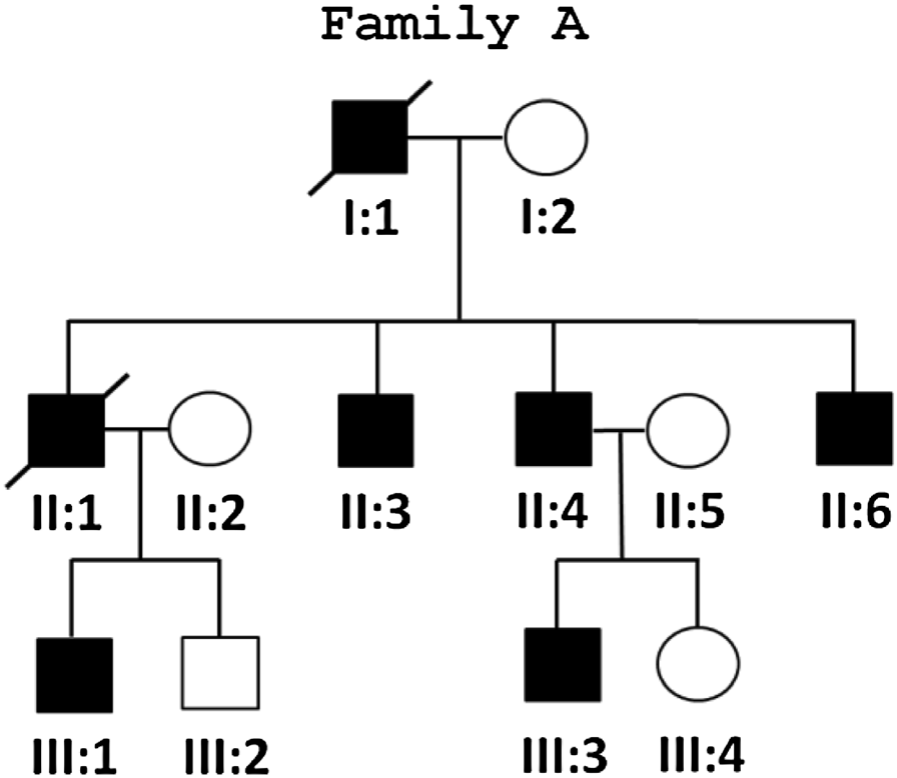
Pedigree of the U.S. family (Family A).

Genomic DNA was extracted from peripheral blood samples according to standard protocols. DNA samples from five affected subjects, II:3, II:4, II:6, III:1, and III:3 of family A (Figure 1), were subjected to whole-exome sequencing (WES). Exon enrichment was performed using the Sureselect Human All Exon capture system (Agilent Technologies Inc, Santa Clara, CA) following the manufacturer protocol. DNA library consisting of paired end reads was sequenced on a Genomic Analyser IIx sequencer (Illumina Inc, San Diego, CA). Library construction, sequence generation, sequence alignment to the reference genome and variant calling were described in detail elsewhere [28,29].

### Mutation screening

Mutation screening by conventional Sanger sequencing was performed in all members of the affected families. In addition, a cohort of 45 unrelated subjects consisting of 11 patients with predominantly proximal weakness, respiratory failure and/or cardiomyopathy, 8 cases with predominantly proximal myopathy, and 26 cases with initial distal muscle involvement subsequently progressing to proximal limb weakness were also screened for *TTN* mutations. The 45 patients have previously been tested for mutations in MFM-associated and other neuromuscular genes (*DES, MYOT, LDB3, CRYAB, BAG3, FLNC,* and *FHL1*) with negative results. Most of the 45 patients originated from North America (U.S. and Canada) and European countries (France, Germany, Poland). Finally, we studied a Spanish patient recently classified as suffering from HMERF on the basis of clinical and pathological data. The screening was done for the following *TTN* mutations associated with HMERF: p.Gly30150Asp identified in the current study, p.Cys30071Arg recently identified in North European families [7,8], and the kinase domain p.Arg32450Trp mutation [5]. Initial screening in the cohort of 45 patients was performed by digesting of amplified exons with restriction enzymes NlaIII, BstNI, and MspI recognizing these mutations, followed by validation of the presence of a *TTN* mutation by using standard Sanger sequencing of amplified *TTN* exons. Primers were designed using Primer3 software (http://frodo.wi.mit.edu/primer3/).

## Results

### Clinical manifestations

*Family A.* Five members of the U.S. family (Figure 1) were studied in detail. They presented between the ages of 13 to 29 with proximal weakness in the lower extremities manifesting with difficulty on stairs or rising up from a squatting position (Table 1). Over the following several years, the weakness spread to distal leg muscles, upper limbs and neck. Within 10 years after the disease onset, older patients (II:1, II:3 and II:6) developed respiratory failure necessitating nocturnal ventilator support and all died before the age of 65 years of respiratory failure. Their father and grandfather suffered from the same disease and died from respiratory failure at ages 52 and 50 years, respectively. None of the patients had cardiac symptoms. Examination of three patients (II:3, II:4, and II:6) at ages 29 to 37 revealed weakness of neck flexors, pectoralis muscles, wrist and finger extensors and flexors, and to a lesser degree intrinsic hand muscles. In the lower limbs, weakness predominated in iliopsoas, hip abductors, ankle and toe dorsiflexors. There was wasting of the trapezius, sternocleidomastoid and calf muscles, but each patient had initially bilateral calf hypertrophy (Table 1). Examination of the two younger patients (III:1 and III:3) within a year of initial symptoms showed weakness in neck flexors, iliopsoas, and ankle dorsiflexors, but no respiratory deficiency (Table 1). Serum CK levels investigated in two patients were found to be normal to two-fold increased. EMG examination in one patient revealed a myogenic recruitment pattern with prominent spontaneous activity at rest.

**Table 1:** Phenotypic characteristics of patients with titin p.Gly30150Asp and p.Cys30071Arg mutations

**Family, Country**	**Family A**					**Family B**	**Family C**
	**U.S., European ancestry**					**Canada, East Indian**	**Spain**
*TTN* Mutation	p.Gly30150Asp					p.Cys30071Arg	
Patient	(II:3)	(II:4)	(II:6)	(III:1)	(III:3)	(Index)	(Index)
Age at onset	29	17	22	13	14	22	36
Age at exam	37	33	29	14	14	26	41
Age at wheelchair	38	-	31	-	-	32	-
Age at respiratory failure	39	-	37	-	-	26	37
Muscles affected at presentation	Proximal LL	Neck, proximal LL	Proximal LL	Proximal LL	Neck, Proximal LL	UL, distal and proximal LL	Proximal LL
Facial muscles	5	5	5	5	5	5	5
Neck flexion	4	4+	4+	4+	5-	5	4
Neck extension	4	5	5	5	5	5	5
Shoulder abduction	4	5	3-	5	5-	4	5
Shoulder adduction	5	4+	4	5	5-	4	5
Elbow flexion	5-	5	5	5	5	5	5
Elbow extension	4+	5	4	5	5	4	5
Wrist flexion	4+	5	4	5	5	4	5
Wrist extension	4+	5	4	5	5	4	4
Finger flexion	4+	5	4	5	5	5	5
Finger extension	4+	5	4	4+	5	4+	4
Intrinsic hand muscles	4+	5	4+	5	5	4+	5
Hip flexion	3-	4+	3-	4	5-	2	3
Hip abduction	4	4	3-	5	5	3	4+
Knee flexion (hamstrings)	4	4+	2	5	5	4	5
Knee extension (quadriceps)	4	4+	3-	5	5	2	4
Ankle dorsiflexion (anterior tibialis)	4	4+	2	4+	4+	3	4
Ankle plantar flexion (gastrocnemius)	4+	4+	2+	5	5	5	5
Toe dorsiflexion	4	4+	2	4+	5	3	2
Toe plantar flexion	4+	4+	4+	5	5	5	5
Gait while ambulant	Waddling	Waddling	Waddling, steppage	Normal	Normal	Steppage	Waddling, steppage
Wasted muscles	Sterno-mastoid, trapezius	Sterno-mastoid, trapezius	Sterno-mastoid, trapezius	none	none	none	none
Bilateral calf hypertrophy	yes	yes	yes	yes	no	yes	no
Pulmonary function	restriction	normal	restriction	normal	normal	Severe restriction	Severe restriction
Dyspnea	yes	no	yes	no	no	yes	yes
Cardiomyopathy	no	no	no	no	no	no	no
CK levels	nt	nt	2-fold-increased	nt	normal	Mildly-increased	2-fold-increased
EMG - Myogenic with spontaneous activity	nt	nt	yes	nt	nt	yes	yes

LL – lower limbs; nt – not tested.

*Family B.*A 26-year-old Native American woman originating from East Indian population of Canada presented at the age of 25 with bilateral foot drop. One year later she developed severe respiratory failure requiring orotracheal intubation. On examination, muscles innervated by cranial nerves were intact. She had bilateral calf hypertrophy and weakness of the deltoids, iliopsoas, hip abductors, quadriceps, knee flexors, and ankle dorsiflexors (Table 1). CK levels were increased 2-fold. Her older sister presented with muscle weakness and respiratory symptoms at the age of 27 years and died at 35 of respiratory failure. Their father, paternal uncle and aunt had similar symptoms and died in their 40s.

*Family C.* A 41-year-old Spanish woman presented at the age of 36 years with difficulty on stairs. Over the following year she experienced dyspnea on exertion and was diagnosed with asthma. On examination, the patient’s facial muscles were intact. She had weakness of neck flexors, iliopsoas, quadriceps, hip abductors, anterior tibialis, and toe extensor (Table 1). Deep tendon reflexes were preserved, and sensation was intact. Her gait was waddling and she had bilateral foot drop. No muscle atrophies or hypertrophies were observed (Table 1). CK levels were 2-fold-increased. Respiratory function test revealed restrictive respiratory insufficiency that was treated with nocturnal ventilation support. The patient’s mother had suffered from slowly progressing predominantly lower limb muscle weakness from the age of 50 years, subsequently developed respiratory insufficiency necessitating ventilatory support at the age of 70, and died two years later from respiratory failure.

### Muscle imaging data

**Figure 2:**
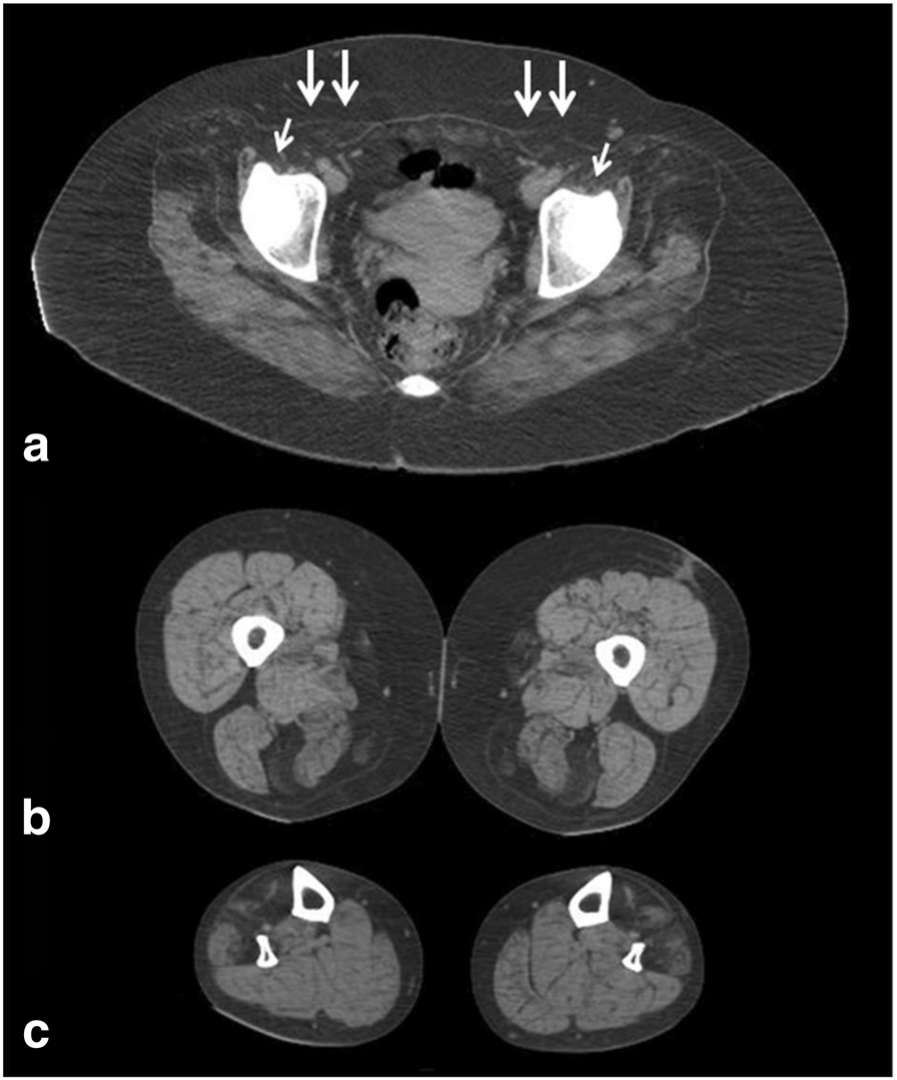
**Muscle CT scans performed in a patient from family C five years after the initial symptoms.** At the pelvic region, the gluteus minimus and medius, and especially the iliopsoas (arrows) and rectus abdominis (double arrows) are severely affected (**a**). Selective involvement of the semitendinosus, sartorius, and gracilis muscles at mid-thigh level (**b**). At the mid-lower-leg level the anterior tibialis and the peroneal group are involved, while muscles of the posterior compartment were well preserved (**c**).

Muscle CT scanning was performed in a patient from family C five years after the initial symptoms. At the pelvic region, the gluteus minimus and medius and especially the iliopsoas and rectus abdominis were severely affected. At mid-thigh, there was selective involvement of the semitendinosus, sartorius, and gracilis muscles. At the mid-lower-leg level, the anterior tibialis and peroneal groups were affected, while muscles of the posterior compartment were well preserved (Figure 2).

### Muscle biopsy analysis

**Figure 3:**
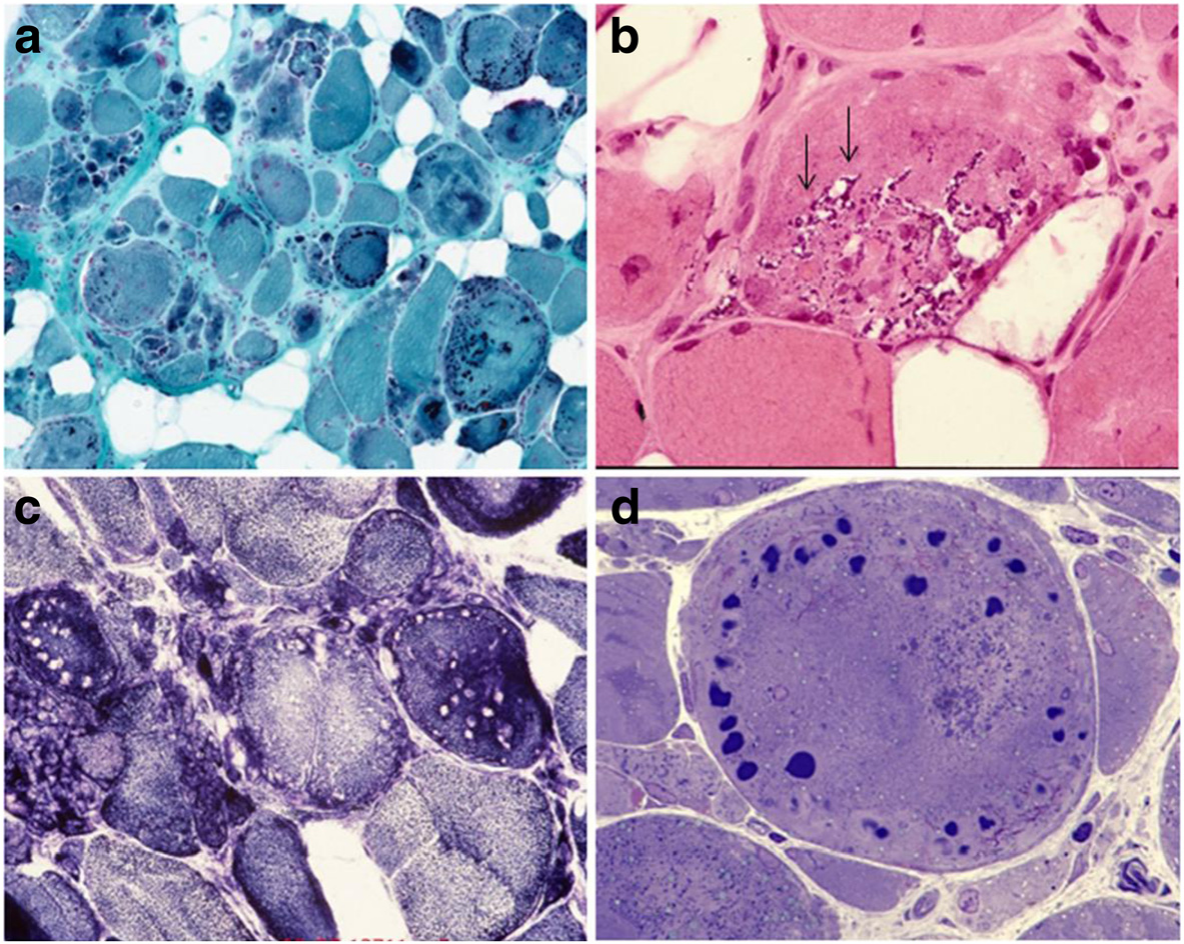
**Histological and histochemical (a, b and c) findings in m. gastrocnemius biopsy of a patient carrying a p.Cys30071Arg *TTN *mutation (family B).** Prominent structural changes with variation of fiber size and areas of fibrofatty tissue replacement; the majority of fibers harbor collections of cytoplasmic bodies along with large diffuse polymorphic accumulations of material (**a**). Some fibers contain rimmed vacuoles (arrows in **b**). Fiber regions occupied by the cytoplasmic bodies are devoid of oxidative activity (**c**). Resin semithin section shows typical cytoplasmic bodies (**d**). Stains: **a**: modified trichrome; **b**: H&E; **c**: NADH; **d**: semithin section stained with toluidine blue.

**Figure 4:**
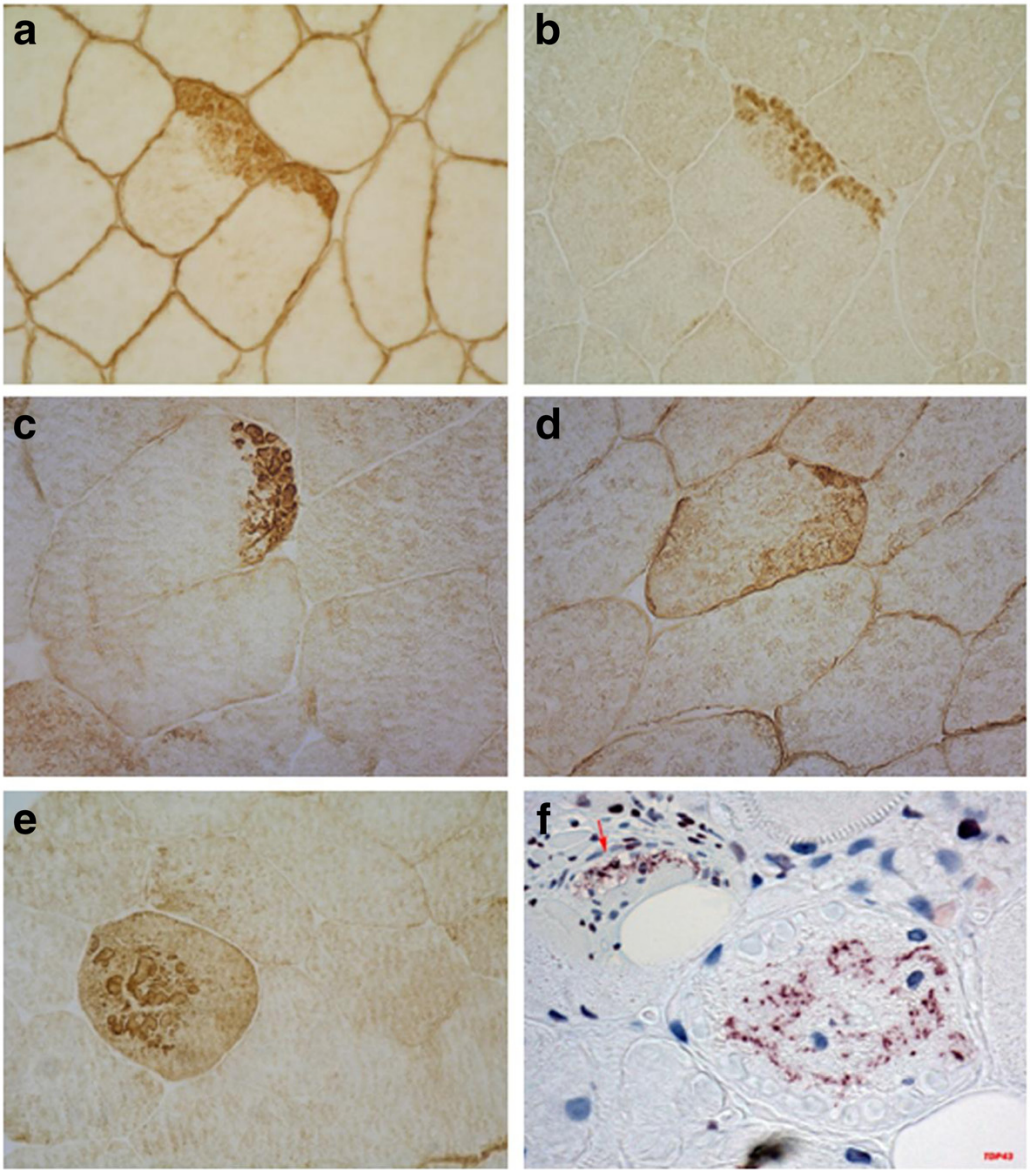
**Immunohistochemical analysis of muscle tissue in patients with *TTN *mutations.** Accumulation of dystrophin (**a**), myotilin (**b**), and filamin C (**c** and **e**) in fiber regions containing cytoplasmic bodies. Desmin (**d**) is focally increased at the periphery of cytoplasmic bodies but not within them. Aggregates of TDP43 (**f**) are seen adjacent to rimmed vacuoles (arrow in f) and around the cytoplasmic bodies.

Myopathological abnormalities were consistent with a myopathy that varied in severity between patients depending on the stage of illness. The most prominent findings were variation in fiber size with atrophic and hypertrophic fibers, increased number of internal nuclei, fiber splitting, increased connective tissue with areas of fibro-fatty tissue replacement and rimmed vacuoles (Figure 3 and Table 2). A characteristic finding in each sample was the presence of small round well defined myofibrillar inclusions of varying size referred to as cytoplasmic bodies. The cytoplasmic bodies were eosinophilic on HE and red, purple or dark blue on modified Gomori trichrome stain, and devoid of ATPase and oxidative enzyme activities. They were at times localized under the sarcolemma. Some severely damaged fibers contained large diffuse polymorphic accumulations of material displaying the same staining properties as the cytoplasmic bodies. Resin semithin sections stained with toluidine blue displayed the cytoplasmic bodies as multiple dark blue accumulations, at times with a peripheral halo. Immunohistochemical analysis revealed accumulation of myotilin, filamin C, dystrophin and αB-crystallin within the cytoplasmic bodies (Figure 4). Desmin was focally increased in the surrounding area but not within the cytoplasmic bodies. TDP43 immunoreactivity was observed adjacent to rimmed vacuoles and in areas around cytoplasmic bodies.

**Table 2:** Pathology features in HMERF patients carrying titin p.Gly30150Asp and p.Cys30071Arg mutations

	**Family A**					**Family B**	**Family C**
*TTN* Mutation	p.Gly30150Asp					p.Cys30071Arg	
Patient	(II:3)	(II:4)	(II:6)	(III:1)	(III:3)	Index	Index
Age at onset	29	17	22	13	14	22	36
Age at biopsy	37	33	29	14	15	25	41
Biopsied muscle	deltoid	deltoid	biceps	quadriceps	quadriceps	gastrocnemius	quadriceps
Variation of fiber size	+++	++	++	+	-	+++	+
Fibro-fatty tissue proliferation	++	+	-	-	-	+++	-
Internal nuclei	+++	+	+	-	-	++	+
Necrosis/phagocytosis	+	-	-	-	-	+	-
Interstitial inflammation	+	-	-	+	-	-	-
Cytoplasmic bodies	+	+	+	++	+	+++	++
Rimmed vacuoles	++	+	++	+	+	++	+
Uneven oxidative enzyme activity	+	+	++	-	-	++	+
Sarcoplasmic tubulofilaments	-	-	-	-	-	+	-
Intranuclear tubulofilaments	-	-	-	-	-	+	-

+ mild; ++ moderate; +++ severe; - not seen.

**Figure 5:**
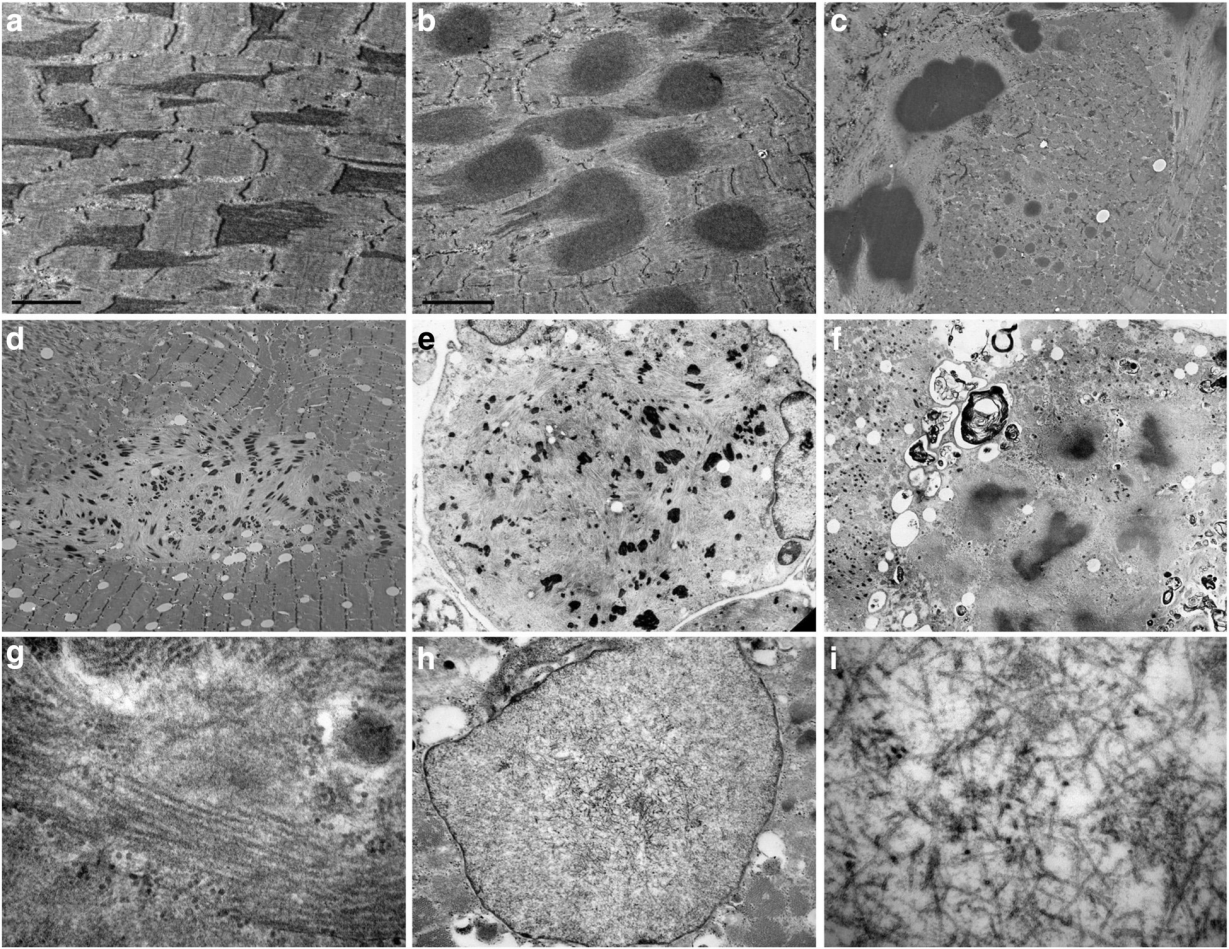
**Ultrastructural images of muscle biopsy in patients with *TTN *p.Cys30071Arg mutation.** Early lesions originate at the Z-lines. Semi-dense filamentous material arises perpendicularly to the Z-lines and extends to the entire sarcomeric length (**a** and **c**). Multiple globular inclusions composed of the same semi-dense filamentous material interspersed between preserved sarcomeres (**b**) or in fibers with sarcomeric disorganization (**c**). A fiber area with focal dissolution of myofibrils and collections of nemaline bodies (**d**). An atrophic fiber with complete sarcomeric destruction is replaced by remnants of filaments and collections of small nemaline bodies (**e**). Typical cytoplasmic bodies surrounded by clear halo and autophagic vacuoles in a severely damaged fiber (**f**). Collections of sarcoplasmic tubulofilaments running in parallel (**g**). Intranuclear tubulofilaments (**h**). Intranuclear tubulofilaments at higher magnification are seen randomly oriented (**i**).

Under electron microscopy, muscle fibers with early changes showed widening and streaming of the Z-lines (Figure 5). There were abundant extensions of semidense filamentous material oriented perpendicularly to the Z-line and comprising the entire sarcomere length. Many fibers contained collections of globular inclusions composed of the same semidense filaments. Typical cytoplasmic bodies with a dense core and a peripheral clear halo were also observed. More severely affected fibers had areas of complete sarcomere disorganization filled by collections of rods, remnants of filaments, cellular debris, and autophagic vacuoles. In a single patient with advanced disease we identified intrasarcoplasmic aggregates of tubulofilaments measuring 16 nm in diameter running in parallel and frequently positioned in areas adjacent to autophagic vacuoles (Figure 5). Also detectable were dispersed and randomly oriented intranuclear thin tubulofilaments of 10 nm.

### Genetic analysis

*Whole exome sequencing results.* We identified the sequence variants from all positions where there were high-quality sequence bases. A number of sequential filtering steps were used to rank variants and to identify potentially pathogenic changes using Most Probable Genotype (MPG) methodology [29]. Non-coding regions and synonymous SNPs were excluded. Missense variants were sorted by the degree of severity of functional disruption prediction using Conserved Domain-based Prediction (CDPred http://research.nhgri.nih.gov/software/CDPred/) [30]. In view of the severity, early age of onset and high penetrance of the disease suggested by the family pedigree, we further restricted our candidates by excluding any variant reported to have occurred in heterozygous or homozygous state in a clinically unaffected adult cohort of 572 subjects (ClinSeq http://www.genome.gov/20519355). The candidate variant position had to have been successfully sequenced to ClinSeq quality standards in at least 100 individuals. Finally, we selected variants shared by five affected members of Family A in heterozygous state, assuming a dominant model.

We have identified a total of 5 variants (Table 3) that fulfilled stringent analysis criteria. Based on information regarding encoded proteins function, we considered variants in *CDC2* and *TTN* as likely candidate mutations for HMERF in family A. The *CDC2* c.175C>T:p.Arg59Cys variant revealed complete co-segregation with the disease phenotype (not shown). CDC2 is a member of serine/threonine kinase family that phosphorylates intermediate filament proteins, including desmin, and an abnormal expression of *CDC2* was found in patients with MFM [31,32]. In light of these reports, we tested expression of CDC2 protein in muscle extracts from an affected subject. Immunoblot analysis with CDC2 specific antibody showed no difference in expression and banding pattern of CDC2 in a patient from Family A (II:4) compared to healthy control subjects (not shown), which contradicts reported data [31]. In addition, the *CDC2* c.175C>T variant is present with a carrier frequency of 0.12% in 4300 publicly available data of exome sequencing in European Americans (NHLBI Exome Sequencing Project/Exome Variant Server http://evs.gs.washington.edu), suggesting that this is a rare polymorphic variant occurring in the background population (*rs*8755).

**Table 3:** Variants identified by whole genome sequencing in Family A

**Chr**	**Genomic Location**	**Mutation type**	**Gene name**	**Ref Allele**	**Var Allele**	**Protein change**	**Deleterious-ness (CPPred)**
**10**	**62214606**	**Nonsyn SNP**	**CDC2**	**C**	**T**	**c.175C>T:p.Arg59Cys**	**−10**
**2**	**179118837**	**Nonsyn SNP**	**TTN**	**C**	**T**	**c.90449G>A:p.Gly30150Asp**	**−9**
**11**	**72144446**	**Nonsyn SNP**	**NY-CO-28, STARD10**	**C**	**T**	**c.578G>A:p.Gly193Asp**	**−6**
**16**	**5080536**	**Nonsyn SNP**	**FAM86A**	**C**	**A**	**c.374G>T:p.Ser125Ile**	**−2**
**18**	**65945866**	**Nonsyn SNP**	**RTTN**	**T**	**C**	**c.3235A>G:p.Ile1079Val**	**3**

These variants (except for the CDC2 variant that is a rare polymorphism designated as *rs*8755) have been submitted to the GenBank; the accession numbers are [GeneBank:JX424570] for the *TTN* variant, [GeneBank:JX424571] for the *NY-CO-28,STARD10* variant, [GeneBank:JX424572] for the *FAM86A* variant, and [GeneBank:JX424573] for the *RTTN* variant.

**Figure 6:**
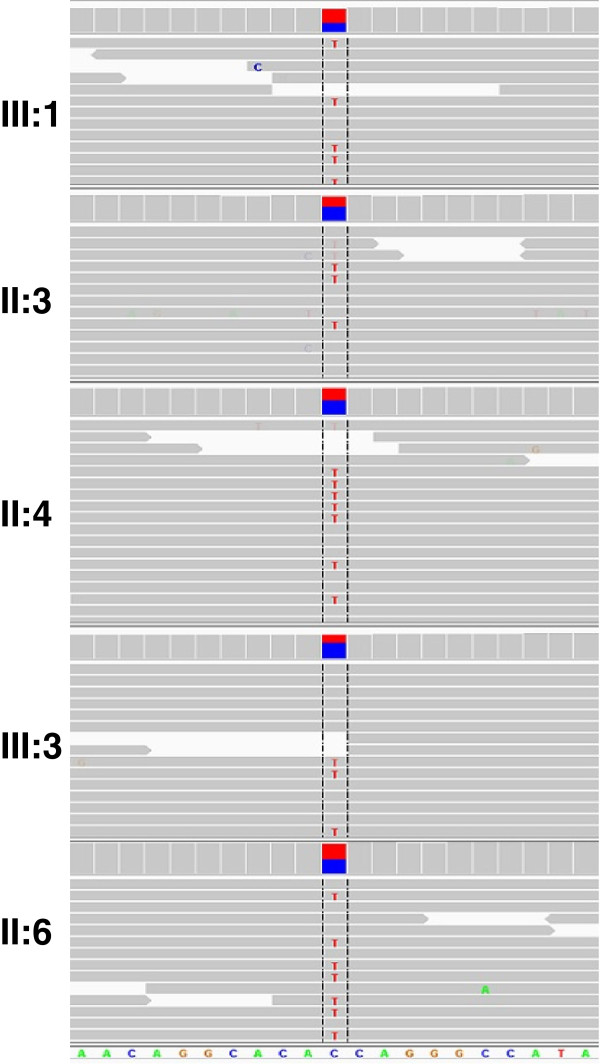
**Screen capture for whole genome sequencing read depth and read alignments using Integrated Genome Viewer (IGV) for the five affected individuals of family A centered at genome position chr2:179,118,837 (red/blue).** Read depths (lines) are scaled in a range of 0–200 for all five individuals. Red cross-lines denote variant reads; the rare variant is present in some but not all reads, indicating heterozygosity.

**Figure 7:**
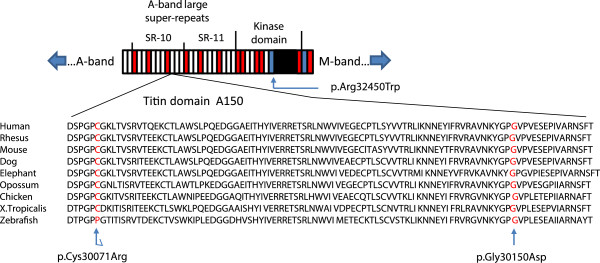
**Schematic representation of super-repeats 10 and 11 (SR-10 and SR-11) of the A-band and the kinase domain of titin.** Fn3 domains are shown in red, Ig-like domains in white and the kinase domain in black. Super-repeat boundaries are indicated by dividing lines. *TTN* mutation p.Gly30150Asp identified in Family A and the p.Cys30071Arg identified in North European families [7,8] have occurred in an FN3 domain of the 10^th^ large super-repeat numbering A150 (according to [33]). The previously reported p.Arg32450Trp mutation [5] is located at the N-terminal helix (alphaR1) of the kinase domain. Bottom: Amino acid conservation (source: http://genome.ucsc.edu) from Zebrafish to Human in the titin domain A150 containing Cys30071 and Gly30150 mutated in HMERF (indicated by arrows).

The second heterozygous variant g.274613G>A (according to database AJ277892) detected in exon 343 of the *TTN* gene was a more convincing candidate. It results in a novel missense mutation c.90674G>A:p.Gly30150Asp (according to databases NP_001243779 and Q8WZ42) that changes a highly conserved neutral glycine to negatively charged aspartic acid. Screen capture of whole exome sequencing (Figure 6) at genome position chr2:179,118,837 shows the presence of the mutation in each affected individual; the variant is present in some but not all reads, indicating heterozygosity. This variant is not reported in ClinSeq 1000 Genomes database or the publicly available data on exome sequencing of 6500 European and African Americans (NHLBI Exome Sequencing Project/Exome variant Server http://evs.gs.washington.edu/EVS). Both the p. Cys30071Arg mutation recently identified in North European families [7,8] and the p.Gly30150Asp mutation of Family A are located in a fibronectin type III (FN3) element within the 10th C-zone super-repeat (Figure 7). In this 10th super-repeat structure(Ig–FN3–FN3–Ig–FN3–FN3–FN3–Ig–*FN3*–FN3–FN3) the mutant fibronectin element is the first of the three C-terminal FN3 repeats and is numbering TTN A150 according to Bucher et al. [33]. FN3-s are known to bind the thick filament of muscle and are highly conserved. Our testing of a structural model of an FN3 titin domain (number A77) solved by X-ray crystallography to 1.65 Å resolution [33] suggest that mutated residues Cys30071 and Gly30150 are located in the side chain that most likely serves as a binding site (not shown).

*Mutation screening.* Both candidate variants, *CDC2* c.175C>T:p.Arg59Cys and *TTN* c.90674G>A:p.Gly30150Asp resulting from WES analysis were confirmed by Sanger sequencing in all affected members of family A. In addition, a cohort of 45 molecularly undiagnosed and unrelated MFM patients was screened for newly identified and previously reported HMERF-associated *TTN* mutations by restriction analysis and additional verification by Sanger sequencing. We identified the p.Cys30071Arg mutation in the index case of a Native American family (Family B). The same p.Cys30071Arg mutation was also detected in the index patient of a Spanish family (Family C) who was suspected of having HMERF based on clinical and pathological data.

## Discussion

After Edström et al. [1] outlined a new disorder based on analysis of patients with an adult-onset proximal myopathy and early respiratory muscle weakness, a number of similar cases were described. The clinical/pathological definitions of this disease later named HMERF were significantly refined and extended [2,3]. Two Swedish families originally reported by Edström et al. were genotyped, the disease-associated locus assigned to chromosome 2q31 in the vicinity of the *TTN* gene [34], and eventually a p.Arg32450Trp mutation in the kinase domain of titin was discovered [5]. Further attempts at finding HMERF-causing *TTN* mutations were hampered by the enormous size of this gene. Next generation sequencing opened up opportunities for new discoveries. Using this technology, the p.Cys30071Arg *TTN* mutation was recently identified as the cause of HMERF in North European families [7,8].

Whole exome sequencing performed on five affected members of Family A showed two variants, c.175C>T:p.Arg59Cys in *CDC2* on chromosome 10q21.1 and c.90674G>A:p.Gly30150Asp in *TTN* on chromosome 2q31 as having the highest deleteriousness scores. Both are segregating with the disease. *CDC2* variant was excluded on the basis that it was not evidently deficient in western analysis and had been recorded in the general population thus suggesting that this variant is rather a rare benign polymorphism (*rs*8755). The *TTN* p.Gly30150Asp change is located in the highly conserved titin A-band and not present in any available SNP database. The final selection of the *TTN* mutation was helped by reports of finding the HMERF-associated p.Cys30071Arg mutation in North European families [7,8]. Screening of 45 additional familial and sporadic patients in which the role of MFM-associated genes have been excluded led to the identification of the *TTN* p.Cys30071Arg mutation in the index patient of family B originating from Native American population; this same mutation was found in a HMERF patient from Spain, indicating that missense mutations in *TTN* are the cause of HMERF in diverse populations.

We compared the disease phenotypes in groups of HMERF patients originating from different populations and carrying various *TTN* mutations (Table 4). Although the number of patients in each column is small for a comprehensive comparison, there is enough data to summarise the basic features of this disease. The pattern of inheritance is autosomal dominant. The age of disease onset is in the teens or the 20-s in most patients; it was at somewhat older age in the U.K. family [2,7]. Pelvic and shoulder girdle weakness was the earliest and predominant sign in every patient, with several exceptions in the family from the U.K. which showed weakness in distal muscles at presentation. In the U.S. family, we observed characteristic sternocleidomastoid/trapezius muscle weakness and atrophy without scapular winging, and calf hypertrophy that was evident before muscle wasting has developed with disease progression. Respiratory function was affected early in the disease: about a third of patients presented with respiratory insufficiency at the disease onset or developed during the first year of illness, and two thirds had moderate respiratory failure requiring ventilation support before the fifth year of illness while they were still ambulant. With disease progression, most of the patients developed weakness in the ankle dorsiflexors. None of the HMERF patients had cardiac involvement, although mutations in *TTN* are known to cause dilated and hypertrophic cardiomyopathy [14]. This may depend on differential expression of tissue-specific titin isoforms. Creatine kinase (CK) levels were normal or slightly elevated. Muscle imaging in our patient showed characteristic and diagnostically significant abnormality in iliopsoas, obturator externus, semitendinosus, gracilis and sartorius muscles on the thigh level and the peroneal group at the mid-lower-leg level, which is in agreement with previous observations [3,8]. Disease outcome for patients with HMERF is one of the worst known for an autosomal dominant adult-onset myopathy because it leads to incapacity, wheelchair dependency, and the need for permanent ventilatory support relatively early in life.

**Table 4:** Comparative clinical characteristics of reported patients with confirmed titin mutations

**Reference**	**Edström et al. 1990**	**Ohlsson et al. 2012**	**Pfeffer et al. 2012**	**This report**	**This report**
***TTN *mutation (TTN domain)**	**p.Arg32450Trp (kinase domain)**	**p.Cys30071Arg (A-band)**	**p.Cys30071Arg (A-band)**	**p.Gly30150Asp (A-band)**	**p.Cys30071Arg (A-band)**
Inheritance pattern	AD	AD	AD	AD	AD
Country	Sweden	Sweden	Britain	U.S.	Spain/Canada
No. of studied patients	7	8	22	5	2
Onset age, mean (range), yrs	24.8	28.8	44.6	19	29
	(14–40)	(18–40)	(22–71)	(13–29)	(22–36)
Gender (woman/man)	5/2	5/3	12/10	(0/5)	2/0
**Distribution of muscle weakness**					
Pelvic girdle	7/7	8/8	15/21	5/5	2/2
Shoulder girdle	7/7	4/7	10/22	3/5	1/2
Neck flexors	7/7	8/8	7/22	5/5	2/2
Trunk muscles	Nr	8/8	Nr	3/5	1/2
Knee flexors	Nr	7/8	7/22	3/5	1/2
Knee extensors	Nr	2/8	6/22	3/5	2/2
Ankle dorsiflexors	2/7	7/8	17/22	5/5	2/2
Plantar flexors	Nr	1/8	5/22	3/5	0/2
Finger extensors	4/7	1/8	4/22	3/5	2/2
Finger flexors	Nr	1/8	3/22	2/5	0/2
Respiratory failure	7/7	8/8	12/22	2/5	2/2
Mildly elevated CK	1/7	1/1	11/19	1/1	2/2
Myopathic EMG	7/7	1/1	11/18	1/1	2/2
**Outcome**					
Wheelchair dependency	Nr	1/8	2/22	2/5	1/2
Ventilation dependency	4/7	4/8	6/22	2/5	2/2
Death before age 65	Nr	1/1	Nr	2/2	1/1

AD – autosomal dominant; Nr – not reported.

The most characteristic abnormalities on muscle biopsies of patients with HMERF are the small round well defined eosinophilic cytoplasmic bodies. Patients with advanced disease show in addition large diffuse polymorphic accumulations of material corresponding to protein aggregates that contain F-actin, dystrophin, myotilin, and filamin C as we determined by immunohistochemistry and as shown in other series of HMERF patients. In agreement with previous observations [4], desmin was focally increased under the sarcolemma and at the periphery of cytoplasmic bodies but not within them. Interestingly, we detected TDP-43 aggregates in the sarcoplasm of several fibers. TDP-43 (TAR-DNA-binding protein-43) is typically found in cytoplasmic inclusions of patients with motor neuron disease and frontotemporal lobar degeneration [35]. TDP-43-stained aggregates were also found in affected muscle fibres in rimmed vacuolar myopathies, including inclusion body myositis, GNE myopathy, myofibrillar myopathies, and oculopharyngeal muscular dystrophy [36-38].

Ultrastructural analysis in two of our patients revealed early disintegration of the Z-disks followed by myofibrillar dissolution and aggregation of degraded filaments. Taken together, the immunohistochemical and ultrastructural features are closely reminiscent of those seen in MFM and based on these morphological characteristics HMERF can be considered a member of the same large group of muscle diseases recently named protein aggregate myopathies (PAMs) [39]. While differential diagnosis of HMERF is solidly based on the presence of cytoplasmic bodies, it needs to be indicated that cytoplasmic bodies observed in HMERF-affected muscles are reminiscent of and should be distinguished from the globular cytoplasmic inclusions seen in muscle biopsies of a fraction of patients with Pompe disease [40]. This is of major importance since early respiratory weakness is also frequently observed in patients suffering from the late-onset Pompe disease [41]. Strong acid phosphatase activity and lack of filaments or any other Z-disc components in Pompe disease structures [40] may help to distinguish them from cytoplasmic bodies found in HMERF. Accumulation of cytoplasmic and occasionally intranuclear tubulofilaments have been noted in patients with MFM, particularly myotilinopathy [42-44] and C-filaminopathy [45,46], demonstrating that the boundaries between HMERF and MFM need to be defined more clearly. Indeed, the combination of proximal and distal weakness and respiratory failure, muscle imaging results and myofibrillar inclusions on muscle histopathology make HMERF similar to desminopathy or alphaB-crystallinopathy. A major distinguishing feature is the lack of cardiac involvement which is seen in the majority of patients with desminopathy or alphaB-crystallinopathy [47] and, importantly, granulofilamentous material which constitutes the ultrastructural hallmark of desminopathy and alphaB-crystallinopathy [27,44,48] is not a feature of HMERF.

There is very limited data on possible consequences of disruptions in FN3 elements within the conserved titin A-band region, although there has been a report of a mutation occurring within FN3 domains in patients with arrhythmogenic right ventricular cardiomyopathy [49]. A three-dimensional structure of an FN3 domain revealed that many of its conserved residues are exposed on the surface of the domain; most likely, they serve as binding sites specifically binding sub-fragment 1 of myosin [20]. Residues replaced by HMERF mutations are known to be highly conserved, specifically the cysteine residues that are the most conserved throughout the entire A-band structure [20]. If the previously proposed structural model of A77 [33] reflects the structure of other A-band FN3 domains, the mutated residues Cys30071 and Gly30150 are located in the side chain that most likely serve as a binding site. Some indications regarding the pathomechanisms in HMERF come from the results of experimental manipulations with FN3 domains that led to an increased rate of molecular aggregation [50].

The p.Arg32450Trp mutation in the kinase domain identified in Swedish families may affect its ability to send signals regarding overextention and other abnormal physiological events in the contracting muscle [51]. Specifically, the mutation disrupts the interaction of titin with NBR1 protein, a ubiquitin-binding scaffold protein, and in turn with p62/SQSTM1 and MURF2. The NRB1/P62 complex plays an important role in autophagic and proteasome degradation of ubiquitinated proteins [5]. The affected muscle shows disrupted myofibrils, NBR1 localization in cytoplasmic bodies, and aggregation of the p62/SQSTM1 complex with myofibrillar proteins. This suggests that the p.Arg32450Trp mutation affects the ability of titin to control muscle protein turnover and through this action disrupts muscle sarcomere maintenance.

## Conclusions

Molecular analysis of patients initially diagnosed as myofibrillar myopathy led to the identification of a novel *TTN* p.Gly30150Asp that is now added to two other known *TTN* mutations causing HMERF. Although there is strong phenotypic similarity between MFM and HMERF, we emphasize distinct clinical/pathological features that can serve as a basis for HMERF diagnosis in patients carrying any of the 3 known *TTN* mutations and originating from diverse world populations. The newly identified p.Gly30150Asp and the other recently discovered p.Cys30071Arg mutation are localized to a side chain of fibronectin type III element A150 of the 10th C-zone super-repeat of titin.

## Competing interests

The authors declare no competing interests.

## Authors’ contributions

CT contributed to writing the manuscript, the study concept and design, the analysis and interpretation of data. MO contributed to the study concept and design, clinical and pathological studies, writing the manuscript and the analysis and interpretation of data. MCD contributed to the study concept and design, clinical and pathological studies, writing the manuscript and the analysis and interpretation of data. KS, JMB, FT, NV, EF contributed to the study concept and design, generating of data, clinical evaluations, pathological studies, and the analysis and interpretation of data. NS contributed to the study concept and design, carried out genetic studies, participated in analysis and interpretation of data. LGG conceived the study, contributed to the study concept and design, writing the manuscript and the analysis and interpretation of data. All authors approved the final version of the manuscript.

## Pre-publication history

The pre-publication history for this paper can be accessed here:

http://www.biomedcentral.com/1471-2377/13/29/prepub
